# Dietary patterns and their socioeconomic factors of adherence among adults in urban Burkina Faso: a cross-sectional study

**DOI:** 10.1186/s41043-023-00451-w

**Published:** 2023-10-10

**Authors:** Konstantin Weil, Issa Coulibaly, Hannah Fuelbert, Alina Herrmann, Roch Modeste Millogo, Ina Danquah

**Affiliations:** 1 Heidelberg Institute of Global Health (HIGH), Heidelberg University Hospital and Medical Faculty, Heidelberg University, Im Neuenheimer Feld 324, 69120 Heidelberg, Germany; 2 Institut Supérieur des Sciences de la Population, Université Joseph Ki-Zerbo, Ouagadougou, Burkina Faso

**Keywords:** Dietary patterns, Nutrition transition, Burkina Faso, Public health

## Abstract

**Background:**

Sub-Saharan African populations undergo a nutrition transition towards diets associated with increased risk for metabolic and cardiovascular diseases. For targeted prevention, we aimed to characterize dietary patterns and determine their sociodemographic factors of adherence.

**Methods:**

We recruited 1,018 adults aged >  = 25 years from two formal and three informal settlements within the Health and Demographic Surveillance System, Ouagadougou, Burkina Faso, between February and April 2021. In a cross-sectional sample, a culture-specific food-propensity questionnaire with 134 food items and a sociodemographic questionnaire were used to collect the data. Exploratory dietary patterns were derived using principal component analysis, and sociodemographic factors of adherence were calculated using multivariable linear regression models.

**Results:**

In this study population (median age: 42 years, interquartile range 21 years; male: 35.7%), the diet relied on starchy foods and other plant-based staples with rare consumption of animal-based products. We identified three dietary patterns, explaining 10.2%, 9.8%, and 8.9% of variation in food intake, respectively: a meat and egg-based pattern associated with younger age, male sex, better education, and economic situation; a fish-based pattern prevailed among women, higher educational levels, and better economic situation; and a starchy food-based was associated with younger age and sharing a home with other adults.

**Conclusions:**

This study population is at an early stage of the nutrition transition and shows low intakes of health-beneficial food groups. Yet, progress along the nutrition transition varies according to age, educational attainment, and economic status. Particularly, younger and well-off people seem to adhere more strongly to diets high in animal-based products. These findings can inform strategies in public health nutrition for sub-Saharan African populations.

**Supplementary Information:**

The online version contains supplementary material available at 10.1186/s41043-023-00451-w.

## Background

Unhealthy diets constitute an important risk factor for morbidity and mortality worldwide. The Global Burden of Disease Study estimates that in 2019, 7.94 million deaths globally were attributable to poor diet, defined as too much intake of unhealthy foods (e.g., salt, red meat, sugar-sweetened beverages) or too little intake of healthy foods (e.g., fruits, vegetables, whole grain products). The majority of these deaths were of cardiovascular nature, such as ischaemic heart disease or stroke. [1]

This risk factor has become more pertinent for sub-Saharan African (SSA) countries in recent decades, as this region is undergoing major shifts in dietary behaviour and faces subsequent health risks. It is widely accepted that many countries in sub-Saharan Africa experience a so-called nutrition transition, characterized by a “modernization” of the diet (e.g. higher intake of refined carbohydrates, processed foods, red meat), while traditional diets (e.g. high intake of grains, vegetables) are less commonly consumed [2]. This change in dietary behaviour leads to an increased risk of obesity and noncommunicable diseases, while the burden of infectious diseases only declines slowly, resulting in a “double burden of disease” [3, 4]. Urbanization forms a major driver of rapid changes in lifestyle, including dietary habits [5]. A commonly used framework to describe stages of the nutrition transition for population groups and corresponding demographic, economic, and health characteristics has been proposed by Popkin [2]. According to this framework, low- and middle-income countries are positioned at various stages of the change from diets of low variety with a high consumption of fibre, starchy foods, but a low consumption of fat and meat (stage 3) towards a diet high in fat, sugar, and processed foods (stage 4), before moving towards a more health-conscious pattern of reduced fat and sugar, but more fruits and vegetables (stage 5). [2]

In sub-Saharan Africa, inhabitants of urban areas are particularly exposed to drastic changes in lifestyle and diet, resulting from rapid economic growth and rural-to-urban migration [6]. This is particularly true for inhabitants of informal settlements. The United Nations Human Settlement Programme estimates that in 2018, at least 238 million people in sub-Saharan Africa lived in informal settlements [7]. Generally speaking, inhabitants of informal settlements face a multitude of health risks, one of which is poor nutrition and less diverse diets [8]. Ouagadougou has been experiencing a massive increase in population size over the last decades, mainly in the form of unplanned growth in the outskirts of the city [9].

While the concept of changes in nutrition in accordance with economic development is widely accepted, data from low-income countries remain scarce in scientific literature, hence little is known about the current state of this transition, for instance in Burkina Faso [4]. Furthermore, existing studies on dietary habits are limited in sample size [10, 11] or focus on specific population groups, such as mothers and children [12, 13], and thus lack representativeness for other contexts.

Traditionally, epidemiological studies examining the relationship between diet and health outcomes or diet and sociodemographic indicators have focused on individual foods or nutrients. However, this does not reflect the complexity and variability of human nutrition. As a result, dietary pattern analysis has emerged as a tool to capture diet more holistically [14]. Principal component analysis (PCA) is one of the most popular techniques to construct dietary patterns empirically and purely data-driven [15, 16]. This exploratory approach is especially useful to characterize population-specific dietary practices in diverse sociocultural contexts. Additionally, characterizing dietary patterns can inform the design of nutrition interventions, and public health messages about dietary patterns might be easier to comprehend than recommendations about single food items or nutrients.

A considerable volume of literature suggests a causal relationship between higher socioeconomic status and a healthier diet (here defined as a diverse diet high in intake of foods such as whole grains, lean meat, fruits, and vegetables, but low in energy-dense and nutrient-poor foods) [17–19]. However, the majority of these studies have been conducted in populations from the global north, not appreciating different social compositions and dietary habits in other contexts. In contrast to high-income countries, in low- and middle-income countries, the opposite effect might be observed, when higher socioeconomic status increases access to, e.g., highly processed, store-bought foods high in saturated fats and sugar, while this access might be limited for people with less resources [20]. Focusing on the link between sociodemographic characteristics and dietary patterns is the basis for informing stakeholders in policy-making and designing target-group specific interventions. Conducting this study in the only urban health and demographic surveillance system in Western Africa ensures representativeness and makes sure that informal settlement dwellers are included.

In summary, there is a lack of representative studies which assess the state of nutrition transition and its links to socioeconomic factors in low-income countries, particularly in SSA. Our study aimed to fill this gap by i) characterizing prevailing dietary patterns and ii) defining the relationships between sociodemographic factors and adherence to different dietary patterns in a representative sample of adults from Ouagadougou, Burkina Faso.

## Methods

### Study design and setting

Between February and April 2021, we conducted this cross-sectional study in Ouagadougou, the capital of Burkina Faso, which has approximately 2.7 million inhabitants. The city is host to the Ouagadougou Health and Demographic Surveillance System (HDSS), covering a contiguous population of about 82,000 people in official neighbourhoods of the city (‘formal’ settlements), as well as unregulated parts in the periphery (‘informal’ settlements). HDSS participants are visited regularly (on average every 10 months) by fieldworkers in their homes to monitor vital events (such as births or death), as well as in- and out-migration. [21]

This manuscript follows the STROBE-nut guideline to provide more transparent reporting (see Additional file 1 for the corresponding checklist) [22].

### Sampling and recruitment

Adults aged ≥ 25 years were eligible for this study. Fieldwork was conducted in accordance with national anti-COVID-19 measures, and personal protective equipment was made available to the fieldworkers.

We based our sample size calculation on the previously observed distribution of energy intake in urban Ghana, measured by the same food-frequency questionnaire [23]. In this previous study, mean energy intake was 2313 kcal/d, and the standard deviation was 668 kcal/d. At 95% confidence interval and an acceptable error of 45 kcal/d, the estimated population size for the present study was 852. Allowing for potential non-participation, we aimed at recruiting 1000 study participants.

To account for unavailable would-be participants and those unwilling to participate, a total of 1945 households with at least one adult aged >  = 25 years were drawn from the HDSS population with equal distribution between formal and informal settlements. Of those households, one adult was randomly selected for the interview. Trained field workers visited the homes and enrolled eligible household members. Upon visiting these households, we met 1,087 eligible subjects (formal settlements: 545, informal: 542). Of these, 93.6% were successfully interviewed (formal settlements: 92.8%, informal: 94.5), for a grand total of 1,018 participants (formal settlements: 506, informal: 512).

**Fig. 1:**
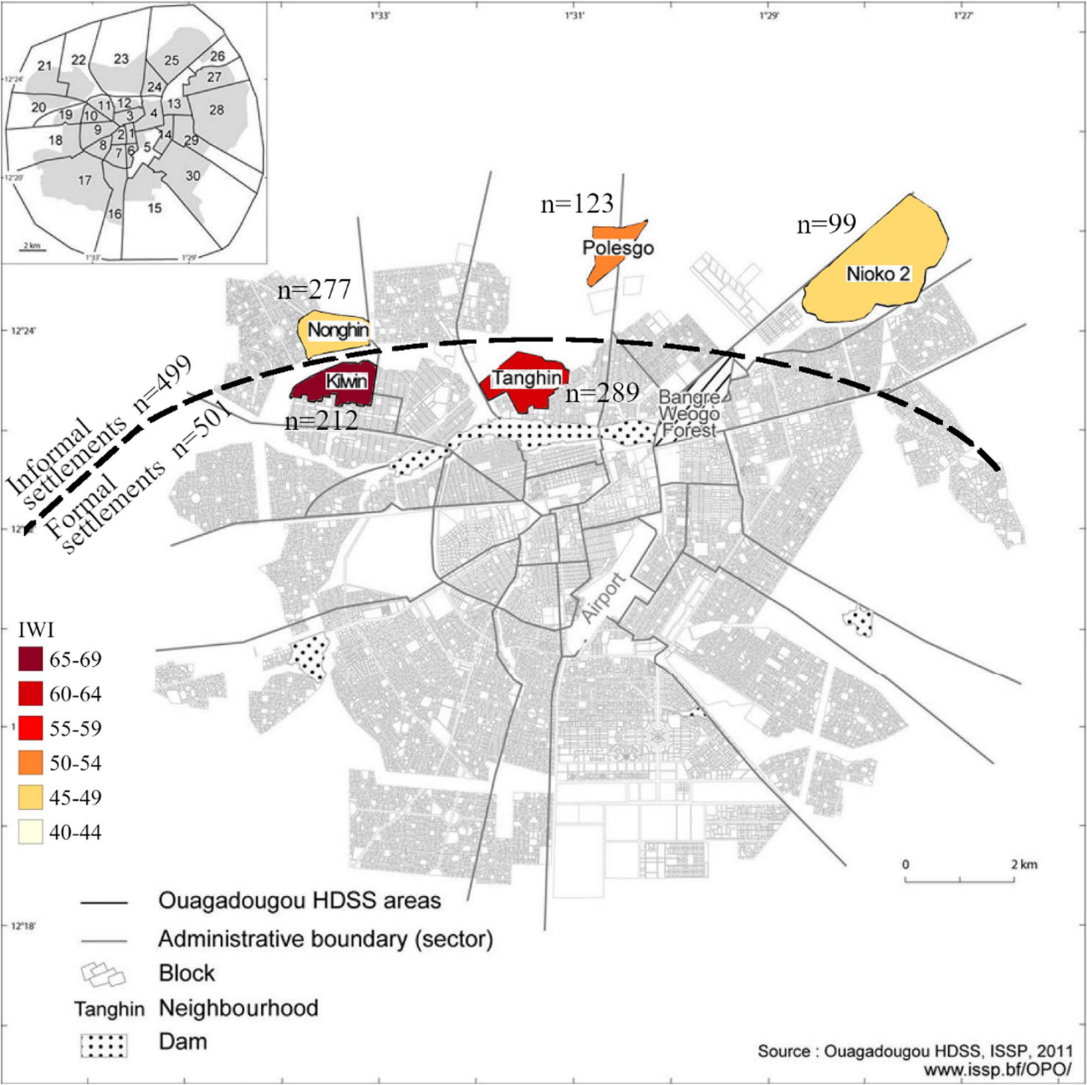
Recruitment sites in Ouagadougou and number of participants. Numbers next to each neighbourhood represent the mean IWI score, also reflected in the colour hue.

Figure 1 shows the locations where the participants were recruited within the Ouagadougou HDSS.

### Dietary assessment

Questionnaires were administered by trained fieldworkers in face-to-face interviews at the homes of the participants. We used tablets and the Survey CTO software (Dobility, Inc., Cambridge, Massachusetts, USA) for data collection and management. Dietary assessments were conducted using the African Food Propensity Questionnaire (AFPQ), a semi-quantitative food-frequency questionnaire that has been adapted from the Ghana-Food Propensity Questionnaire [24]. The AFPQ queries for the intake frequencies of 134 food items in pre-defined portion sizes over the last 12 months. To ensure a common understanding of portion sizes among participants, we showed standardized portion sizes using pictures of common household measures, such as table spoons or cups.

We used the West African Food Composition Database [25] and the German Nutrient Database (BLS 3.01) (2010) to calculate the intakes of energy (kcal per day), as well as macronutrients (energy%) and micronutrients (grams per day).

The original AFPQ and sociodemographic questionnaire were in English. Translation into French was done by a bilingual researcher at the institute, and back-translation was performed to ensure accuracy of the translation.

### Assessment of demographic and socioeconomic factors

During the face-to-face interviews, field workers collected demographic and socioeconomic data. Demographic data comprised age (years), sex (male, female), and self-reported ethnic group (10 categories). Socioeconomic data included educational level (none, primary, junior secondary, senior secondary, vocational training, tertiary), employment status (employed, retired, unemployed, unable to work, on social benefits, full-time homemaker, student), occupation (subsistence farmer, commercial farmer, casual worker, merchant/vendor, craftsperson, civil servant, other), marital status (married, cohabitating, single, divorced/separated, widowed), number of people living in the household, and information, whether the respondents were living together with their partner/toddler/child/parents/in-laws/other adults, or if they were living in a retirement home. Please note that we use the term “homemaker” as an answer option in the employment variable for lack of a better term, although the true workload though additional activities outside the home might not be reflected by it.

Further data for the individual households were available from previous HDSS surveys (2019–2020). Those included information on the source of energy available (gas, firewood, coal), access to electricity/water, floor quality, the type of toiled used, and availability of household items (phone, bicycle, car, television, refrigerator).

### Missing data handling

In total, 1018 questionnaires were completed successfully. After the initial data quality checks, 5% of the participants with the most extreme values (2.5% with the lowest and 2.5% with the highest values) for energy intake (kcal per day) were revisited to confirm the accuracy of their answers. Of those 50 study subjects, 39 were met at home and had their interviews retaken in the same manner as in the first round. Because food intake data reported during the second visit were much more plausible, these data were used in the present analysis.

**Fig. 2:**
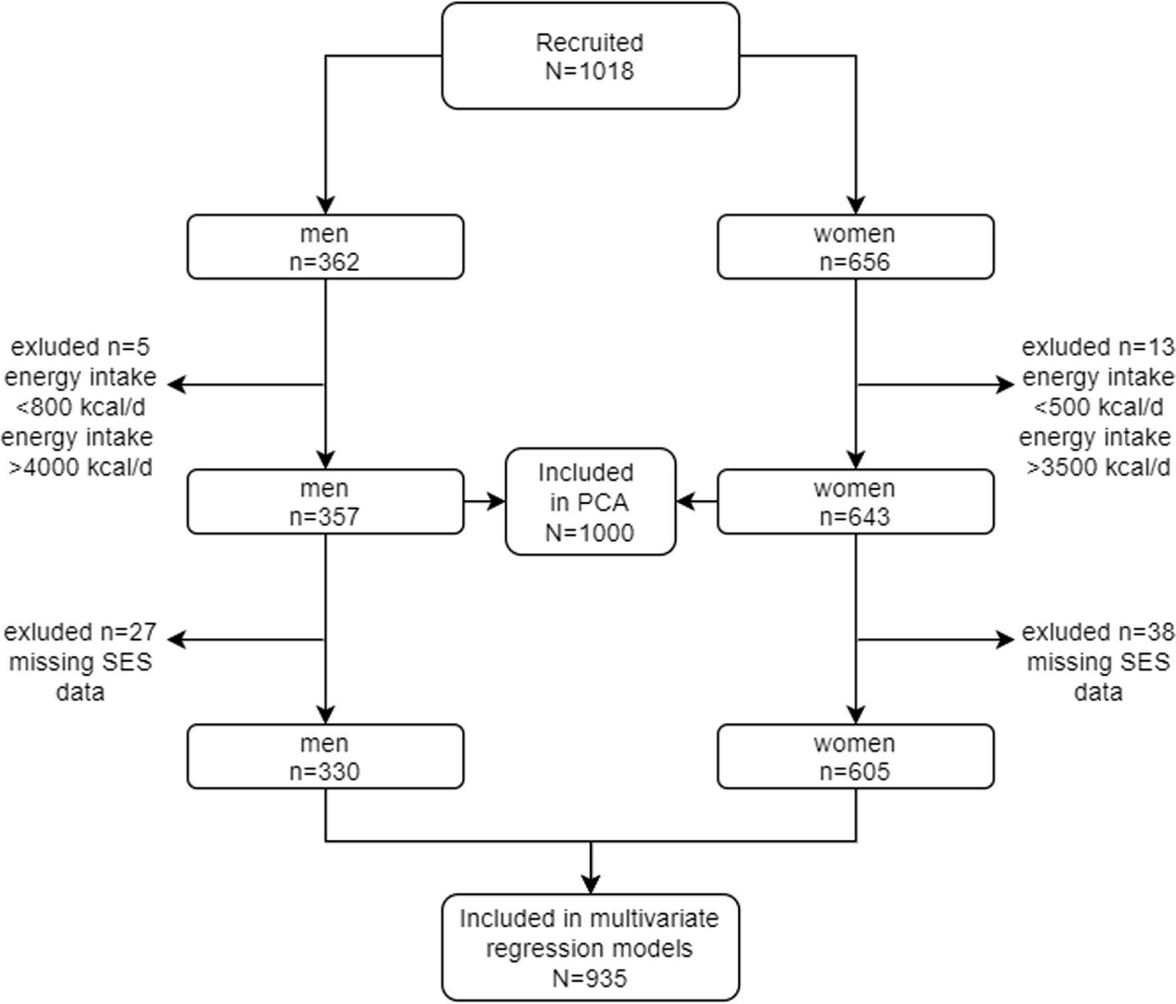
Flowchart of participants included in the analysis and reasons for exclusion. PCA = principal component analysis, SES = socioeconomic status.

In this nutrition survey, we did not perform anthropometric assessments. To approximate energy expenditure, we used literature-based, fixed energy intake cut-offs for men and women as a criterion for data plausibility [26]. For men, we applied plausible ranges of energy intake between 800 kcal and 4,000 kcal; for women, this range was 500 kcal and 3,500 kcal. Figure 2 shows the exclusion of participants based on implausible or missing data. Of the recruited 1018 participants, 5 men and 13 women had implausible values for energy intake. Thus, 1,000 participants were included in principal component analysis and descriptive statistics. Also, there were 27 men and 38 women who had missing data on demographic or socioeconomic variables. Hence, the final analytical sample size was 935 participants, used in the calculation of the International Wealth Index and in the regression models.

## Data analysis

For the characteristics of the study population and the distribution of food intake, we calculated proportions (%) for categorical data, medians and interquartile ranges for non-normally distributed data, and means and standard deviations (SD) for normally distributed data. As a summary variable for measuring the economic status of the households, we used the International Wealth Index (IWI) according to [27], ranging from 0–100 points. Higher score points reflect better access to infrastructure and possessing more household assets. Base variables for the calculation of the score were: household possession (yes/no) of phone/bicycle/car/television/fridge, and whether the household had access to electricity. Floor quality, water quality, and type of toilet were also considered. To test significance of differences in IWI scores between settlement types, we used an unpaired t test with a significance level of 0.05.

To characterize dietary intake, we grouped 134 food items into 30 food categories based on their nutrient profiles and culinary use (Table 1). Using common household measures and portion sizes, we calculated the intake in grams per day for each food group, using the West African Food Composition Table [25]. We present measures of central tendency (median) and dispersion (interquartile range) in the form of bar charts.

**Table 1:** Collapsing of food items of the African Food Propensity Questionnaire (AFPQ) into 30 food groups

Food group	Food items
Whole Grain Bread and Cereals	Muesli/Cereals, Other grains
White Bread and Cereals	Bread/Rolls/Buns, Hot cereal porridge
Sweet Spreads	Jam
Dairy Products	Cocoa milk, Milk, Yoghurt, Sour milk, Flavoured yoghurt, Quark, Soft cheese, Semi firm cheese, Mozzarella, Cream cheese, Butter, Whipped cream
Fruit	Apple/Pear(fresh), Orange, Banana, Plum, Strawberries/Cherries, Watermelon, Mango/Pawpaw, Berries, Grapes, Stewed fruit
Nuts and Seeds	Dried fruit, Nuts/Almonds, Seeds
Roots and Tubers	Plantain, Cassava, Yam, Plantain dough
Potatoes	Potatoes, Pan fried potatoes, Sweet potatoes
Maize-based Foods	Maize dough, Fermented maize
Vegetables	Carrots, Tomatoes, Lettuce/Endive, Cucumber, Peppers, Leaves/Spinach, Cooked white cabbage, Eggplant, Beans, Garlic(raw), Garlic(cooked), Onions(raw), Onions(cooked)
Legumes	Groundnut soup, Legumes, Lentil/pea/bean soup
Vegetable Soups, Stew, and Sauces	Palmnut soup, Green stew, Tomato sauce/stew, Vegetable soup
Rice, Pasta, and Corn	Rice, Pasta/Noodles/Macaroni, Grilled corn
Egg	Egg
Red Meat	Beef, Goat, Pork, Bush meat, Intestine
Poultry	Poultry
Processed Meat	Meatballs, Fried sausage, Boiled sausage, Dry/cured meat, Salami, Jagdwurst/Bologna, Liverwurst
Fish	Fatty fish, Lean fish, Fish preparations, Shellfish
Meaty Mixed Dishes	Lasagna/Pizza, Mixed dishes with meat
Vegetarian Mixed Dishes	Mixed dishes without meat, Tofu
Cakes and Sweets	Tart pie, Yeast cake/pastry, Cake with cream, Cookies/Biscuits, Chocolate, Sweets/Candy
Coffee and Tea	Regular coffee, Decaffeinated coffee, Tea(black/green), Tea(fruit/herbal)
Alcoholic Beverages	Regular beer, Wine, Liquor/Spirits
Sodas and Juices	Non-alcoholic beer, Sodas/Soft drinks, Light soft drinks, Fruit juice, Fruit nectar, Vegetable juice
Palm Oil	Palmnut oil
Olive Oil	Olive oil
Other Oils	Other oils, Peanut butter
Margarine	Regular margarine, Fat-reduced margarine
Cooking Fats	Cooking fats
Condiments	Ketchup, Mayonnaise dressing, Cream dressing, Yoghurt dressing, Vinegar dressing, Other sauces

To find combinations of food categories that were often eaten together, we applied principal component analysis (PCA) using the PROC FACTOR procedure in SAS/STAT® 9.4 (SAS Institute Inc., Cary, NC, USA). We retained factors based on the Eigenvalue criterion > 1, the scree plot, and the interpretability of the pattern scores. We applied the varimax rotation to ensure that the extracted factors remain uncorrelated. Factors were labelled and interpreted as dietary patterns based on the food groups with the highest factor loadings (|correlation coefficient|> 0.4). Participants were allocated a score for each factor based on their individual intake and respective factor loading of the food groups. Factor scores were standardized to have a mean of 0 and an SD of 1.

The robustness of factors was tested by applying PCA to random-split samples, and comparing the resulting pattern scores and their factor loadings with each other. We also ran PCA separately for formal and informal settlements to test the stability of pattern scores.

For the description of factors of adherence, we constructed quintiles of the identified pattern scores and calculated the distribution of demographic and socioeconomic variables across these quintiles. We calculated p-values for trend using the Jonckheere–Terpstra test (non-normally distributed continuous variables), linear regression (normally distributed variables), the Cochran–Armitage trend test (binary variables), and the Cochran–Mantel–Haenszel test for categorical variables. Variables that seemed to have a linear trend across the quintiles of the pattern scores were subjected to correlation analysis to identify intercorrelation. For the following regression models, we have removed variables that showed a moderate to strong correlation with other similar variables (Cramér’s V > 0.3) [28], such as occupation and employment, in order to make the regression models more parsimonious and improve interpretability.

For the associations of demographic and socioeconomic factors with the identified dietary pattern scores, we calculated beta coefficients per one-unit increase, their 95% confidence intervals (CIs), p-values, and the explained variance in the outcome variable (R^2^). We constructed multivariate linear regression models for each dietary pattern with the following set of independent variables: age, sex, education level, employment status, occupation, International Wealth Index, marital status, and living together with a toddler/in-laws/other adults.

## Results

### Characteristics of the study population

Figure 1 provides an overview of the study area with the number of participants from each neighbourhood. The demographic and socioeconomic characteristics of the total study population and by settlement type are shown in Table 2.

**Table 2:** Demographic and socioeconomic characteristics of 1000 adults living in Ouagadougou, Burkina Faso

	*n*	Total	Type of settlement	
			Formal	Informal
N		1000	501	499
Age (years)	1000	42 (21)	47 (25)	39 (15)
Sex	1000			
Male (%)		35.7	36.7	34.7
Female (%)		64.3	63.3	65.3
Ethnic group	1000			
Bobo (%)		0.6	0.8	0.4
Dioula (%)		0.2	0.2	0.2
Fulani (%)		1.8	3.0	0.6
Gourmantche (%)		0.4	0.6	0.2
Gourounsi (%)		1.0	0.8	1.2
Lobi (%)		0.4	0.4	0.4
Mossi (%)		92.0	88.8	95.2
Other (%)		1.4	2.4	0.4
Bissa (%)		1.0	1.6	0.4
Samo (%)		1.2	1.4	1.0
International Wealth Index	935	56.9 ± 16.5	65.5 ± 14.9	47.5 ± 12.5
Education level	1000			
none (%)		57.3	50.9	63.7
Primary (years 1–6) (%)		20.3	19.4	21.2
Limited secondary (years 7–10) (%)		13.8	15.4	12.2
Advanced secondary (years 11–13) (%)		5.8	9.4	2.2
Apprenticeship training (%)		0.4	0.8	0
University (%)		2.4	4.2	0.6
Employment status	1000			
Paid employee (%)		25.1	29.7	20.4
Retired (%)		4.7	8.8	0.6
Unemployed, looking for work (%)		29.0	25.0	33.1
Unable to work (%)		5.2	6.0	4.4
On social benefits (%)		0.1	0	0.2
Full-time homemaker (%)		34.3	28.1	40.5
Student (%)		1.6	2.4	0.8
Occupation	1000			
Subsistence farmer (%)		2.7	3.4	2.0
Commercial farmer (%)		0.8	0.4	1.2
Casual worker (%)		9.4	8.2	10.6
Businessperson, merchant, vendor (%)		45.2	37.7	52.7
Craftsperson (%)		6.3	4.8	7.8
Civil servant (%)		8.0	11.8	4.2
Other (%)		27.6	33.7	21.4
Marital status	1000			
Married/registered (%)		55.3	58.3	52.3
Cohabitating (%)		22.3	14.4	30.3
Single (%)		6.1	8.4	3.8
Divorced or separated (%)		2.8	1.8	3.8
Widowed (%)		13.5	17.2	9.8
Number of people living in the household	1000	6 (4)	6 (4)	5 (3)
Main source of energy used for cooking	938			
Not applicable (%)		1.9	2.1	1.8
Gas (%)		37.6	45.5	29.2
Firewood (simple format) (%)		20.0	17.5	22.8
Firewood (improved format) (%)		14.4	13.8	15.0
Coal (simple format) (%)		15.0	10.5	19.9
Coal (improved format) (%)		10.8	10.7	10.8
Other (%)		0.2	0	0.4
Number of refrigerators	938	0 (0)	0 (0)	0 (0)

Data presented as means ± standard deviation, medians (IQR) or percentages. The International Wealth Index (Smits and Steendijk 2015) reflects access to basic infrastructure and possession of specific household and luxury items

Most of the respondents were female (64.3%). The median age in the sample was 42 (IQR: 21) years; this number was 47 years (IQR: 25 years) in the formal settlements and 39 years (IQR: 15 years) in the informal settlements. The majority of participants identified as Mossi, the biggest ethnic group in Burkina Faso. Most people had not completed any formal education (51% to 64%). Also, the majority of participants were not in a stable employment situation, either being unemployed (29.0%) or being a full-time homemaker (34.3%, formal: 28.1%, informal: 40.5%). The median number of household members was 6 (IQR: 4).

The mean IWI score was 56.9 ± 16.5, being 65.5 ± 14.9 in the formal settlement and 47.5 ± 12.5 in the informal settlements. This indicates a significant difference in IWI scores between the settlement types (p < 0.0001). The mean IWI scores for each neighbourhood are shown in Fig. 1 (colour shading).

### Food groups and nutrient intakes

**Fig. 3:**
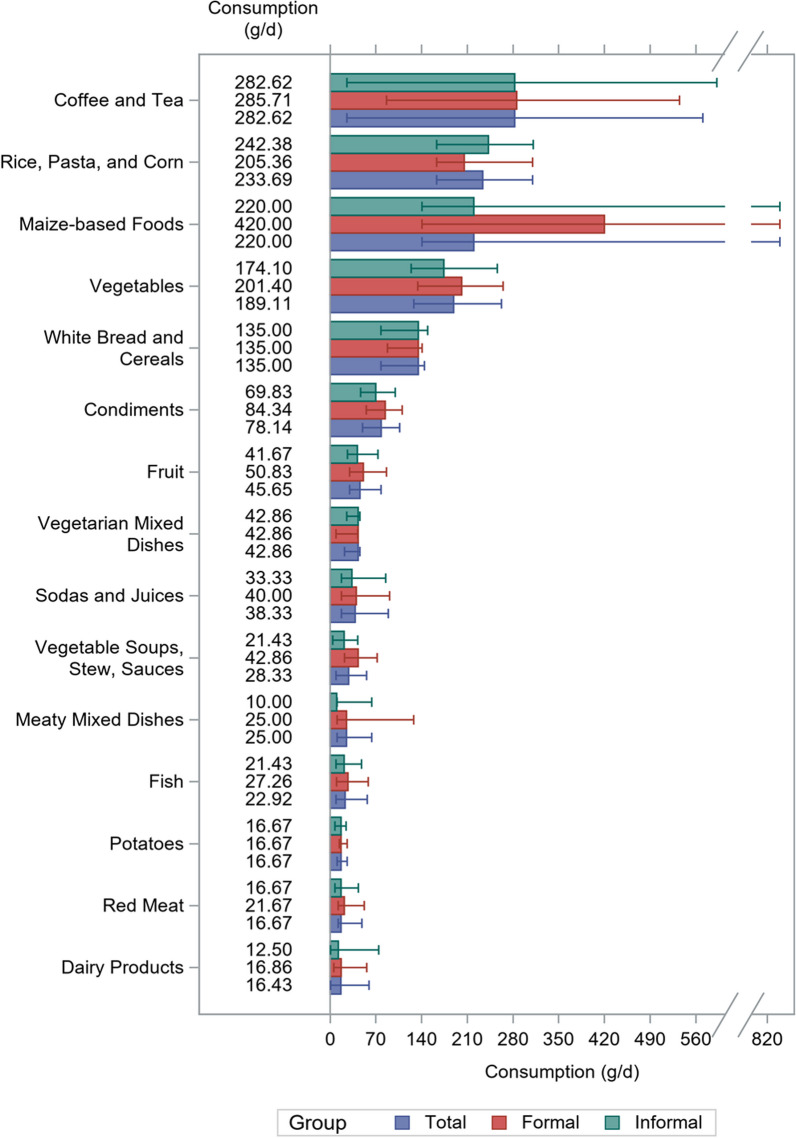
Median daily consumption of food groups (> 15 g/d) among 1000 adults in Ouagadougou

**Fig. 4:**
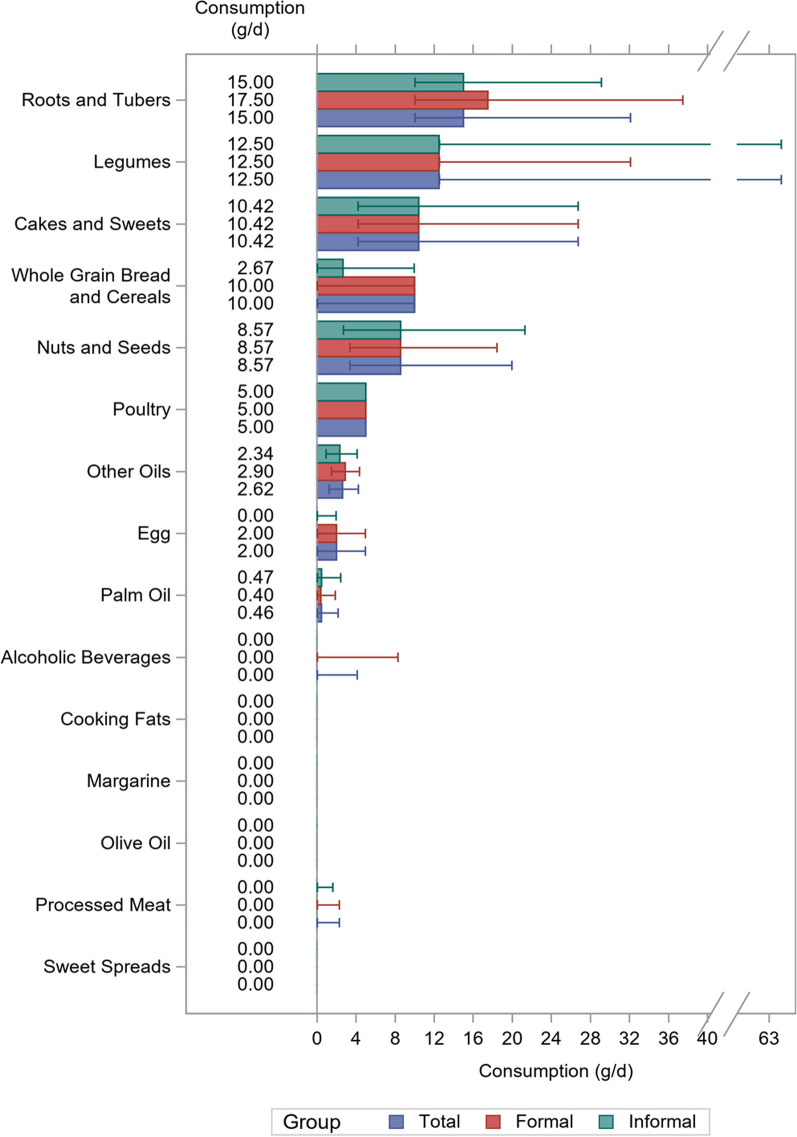
Median daily consumption of food groups (≤ 15 g/d) among 1000 adults in Ouagadougou

Figures 3 and 4 show the median consumption of 30 food categories in g/d for the total study population and by settlement type. The highest intake was seen for coffee and tea; rice, pasta and corn; maize-based foods; vegetables; and white bread and cereals (Fig. 3). In contrast, alcoholic beverages, margarine, cooking fats, olive oil, processed meat, and sweet spreads were rarely consumed. In general, animal-based products (e.g., egg, poultry, dairy products, red meat, and fish) were rarely consumed. Table 3 shows the energy intake and nutrient consumption for the total study population, by settlement type and by sex. The median energy intake in the whole sample was 1834 kcal/d (IQR: 908), amounting to 1958 kcal/d (IQR: 875) in formal settlements, and to 1731 kcal/d (IQR: 884) in informal settlements. Carbohydrates, total fats, and protein accounted for 62%, 24%, and 13% of energy intake, respectively. Mean fibre intake was 25 ± 17 g/d, and the ratio of saturated versus unsaturated fats was 0.81 ± 0.27.

**Table 3:** Daily energy, macronutrient intake, and dietary pattern scores in 1000 adults from Ouagadougou

	Total	Type of settlement			
		Formal		Informal	
		Sex		Sex	
		Male	Female	Male	Female
*n*	1000	184	317	173	326
Total EI (kcal day^−1^)	1834.13 (908.08)	2095.73 (862.71)	1901.71 (825.65)	1775.46 (831.47)	1673.7 (886.39)
CHO (% energy)	62.13 (16.74)	62.59 (13.82)	61.26 (17.5)	61.16 (17.9)	62.8 (17.21)
Fat (% energy)	24.34 (13.99)	23.79 (12.9)	25.71 (14.38)	24.34 (14.88)	24.09 (14.35)
Protein (g day^−1^)	61.12 (32.39)	65.18 (34.20)	64.65 (34.20)	58.69 (37.11)	55.77 (31.07)
Protein (% energy)	12.61 (3.12)	12.43 (2.76)	12.73 (3.51)	12.7 (2.76)	12.55 (3.06)
Alcohol (g day^−1^)	0.00 (0.47)	0 (1.31)	0 (0.14)	0 (1.5)	0 (0.06)
Cholesterol (g day^−1^)	0.13 (0.12)	0.15 (0.14)	0.14 (0.12)	0.13 (0.12)	0.11 (0.12)
MUFA (g day^−1^)	14.41 (11.14)	15.29 (11.2)	15.25 (10.56)	13.65 (11.61)	12.88 (11.15)
PUFA (g day^−1^)	6.69 (4.69)	7.11 (4.38)	7.12 (4.09)	6.03 (5.13)	6.41 (5.03)
SFA (g day^−1^)	16.67 (11.33)	18.36 (12.55)	17.97 (10.96)	16.27 (11.18)	15.22 (10.68)
SFA/UFA	0.81 (0.27)	0.82 (0.29)	0.8 (0.25)	0.84 (0.31)	0.8 (0.28)
Fibre (g day^−1^)	25.72 (17.14)	31.74 (15.95)	26.41 (16.71)	22.1 (15.39)	22.74 (14.73)
Meat and egg-based dietary pattern score	0.00 (1.00)	0.34 (1.09)	0.1 (1.13)	0.15 (0.87)	− 0.37 (0.73)
Fish-based dietary pattern score	0.00 (1.00)	− 0.09 (1.02)	0.08 (0.97)	− 0.12 (0.98)	0.03 (1.02)
Starchy food-based dietary pattern score	0.00 (1.00)	0.12 (1.05)	0.02 (1.02)	− 0.13 (0.95)	− 0.01 (0.97)

Nutrients are presented as median (IQR). EI = ”Energy intake”. CHO = ”Carbohydrates”. MUFA = ”Monounsaturated fatty acids”. PUFA = ”Polyunsaturated fatty acids”. SFA = ”Saturated fatty acids”. UFA = ”Unsaturated fatty acids”. Pattern scores (last three rows) are presented as mean (standard deviation). Per definition the mean of pattern scores derived by principal component analysis is set to 0 and the standard deviation is set to 1 for the total of the sample. Positive scores signify a comparatively higher adherence to this pattern (compared to the total sample), and negative scores a lower adherence

### Dietary patterns and their factors of adherence

We retained three dietary pattern scores from PCA that explained 29% of the total variation in food group intake. Table 4 shows the dietary patterns and their rotated factor loadings for 30 food groups.

**Table 4:** Dietary pattern scores derived by principal component analysis and rotated factor loadings of food groups

Food group	Meat and egg-based dietary pattern	Fish-based dietary pattern	Starchy food-based dietary pattern
Poultry	**62**	− 3	16
Meaty Mixed Dishes	**61**	**50**	3
Red Meat	**54**	**55**	7
Egg	**54**	11	14
Potatoes	**45**	− 2	**42**
Processed Meat	**42**	19	6
Dairy Products	38	− 21	− 5
Fruit	38	− 8	**43**
Condiments	36	**63**	**46**
Sodas and Juices	36	7	3
Vegetables	32	**47**	**46**
Coffee and Tea	30	− 39	6
Olive Oil	24	0	7
Whole Grain Bread and Cereals	24	5	21
Roots and Tubers	23	-2	**48**
Alcoholic Beverages	20	4	− 5
Margarine	17	8	− 8
Vegetable Soups, Stew, Sauces	12	21	− 10
Cooking Fats	12	− 4	− 2
White Bread and Cereals	12	− 7	21
Palm Oil	8	**70**	− 29
Fish	5	**71**	− 3
Rice, Pasta, and Corn	3	− 25	**61**
Nuts and Seeds	3	**44**	**44**
Sweet Spreads	1	−2	30
Cakes and Sweets	− 4	27	**44**
Other Oils	− 11	27	37
Legumes	− 15	− 1	**43**
Maize-based Foods	− 35	− 23	**47**
Vegetarian Mixed Dishes	− 35	− 12	0
Total variance explained by factor (%):	10.15	9.82	8.87

Factor loadings are multiplied by 100 and rounded to the next integer. Loadings >  = 40 and <  = -40 are printed bold

The first dietary pattern explained 10.2% of the variation in food group intake and was characterized by high intakes of poultry (0.62), meaty mixed dishes (0.61), red meat (0.54), eggs (0.54), potatoes (0.45), and processed meat (0.42). Therefore, we labelled it as “meat and egg-based dietary pattern”.

The second dietary pattern explained 9.8% of the total variation in food group intake and was characterized by frequent consumption of fish (0.71), palm oil (0.70), condiments (0.63), red meat (0.55), meaty mixed dishes (0.50), vegetables (0.47), and nuts and seeds (0.44). Accordingly, we named this the “fish-based dietary pattern”.

Finally, the third dietary pattern explained 8.9% of the total variation in food group intake and had positive correlations with rice, pasta, and corn (0.61), roots and tubers (0.48), maize-based foods (0.47), condiments (0.46), vegetables (0.46), nuts and seeds (0.44), cakes and sweets (0.44), legumes (0.43), fruits (0.43), and potatoes (0.42). It was labelled “starchy food-based dietary pattern”.

The last four rows of Table 5 show total energy (kcal) and macronutrient (% energy) intake across different quintiles of dietary pattern scores, thus showing the trend of these intakes for increasing adherence to the respective dietary pattern. The meat and egg-based dietary pattern showed decreasing carbohydrate intake (quintile 1: 66%, quintile 5: 57%), and increasing protein intake (quintile 1: 12%, quintile 5: 14%) with increasing adherence to the pattern. The fish-based dietary pattern, in contrast, exhibited decreasing carbohydrate intake (quintile 1: 71%, quintile 5: 46%), increasing fat intake (quintile 1: 17%, quintile 5: 37%), and increasing protein intake (quintile 1: 11%, quintile 5: 16%). The starchy food-based pattern showed increasing total energy intake (quintile 1: 1321 kcal, quintile 5: 2544 kcal) with increasing adherence to the pattern.

**Table 5:** Characteristics across quintiles of the dietary pattern scores in 935 adults from Ouagadougou

	Meat and egg-based dietary pattern				Fish-based dietary pattern				Starchy food-based dietary pattern			
	Q1	Q3	Q5	*p*-trend	Q1	Q3	Q5	*p*-trend	Q1	Q3	Q5	*p*-trend
Age(years)	45(26)	42(21)	39(17)	< .0001	40(17)	42(22)	43(20)	0.1296	47(22)	41(20)	40(21)	< .0001
Sex (male)	19	34	49	< .0001	46	33	30	0.0067	36	36	39	0.8465
Ethnic group				0.4584				0.2590				0.8758
Bobo	1	1	1		1	0	0		0	1	1	
Dioula	0	0	1		0	0	1		0	0	1	
Fulani	1	3	3		1	1	2		3	2	1	
Gourmantche	0	1	1		1	1	0		1	1	1	
Gourounsi	0	1	1		1	2	1		0	2	2	
Lobi	0	1	1		0	0	2		0	0	1	
Mossi	96	92	90		92	94	92		94	92	91	
Other	1	2	1		2	1	1		2	1	2	
Bissa	2	0	1		1	2	1		0	1	2	
Samo	0	1	2		2	0	2		1	2	1	
International Wealth Index	49.7 ± 14.8	56.6 ± 15.9	64.9 ± 16.8	< .0001	54.6 ± 17.5	56.6 ± 16.4	59.6 ± 16.0	0.0008	55.4 ± 17.8	56.7 ± 15.5	59.5 ± 16.4	0.0008
Education level				< .0001				0.0008				0.0100
None	79	64	27		50	64	50		64	54	52	
Primary (years 1–6)	11	20	23		24	15	20		16	22	18	
Limited secondary (years 7–10)	8	12	26		15	15	20		16	14	15	
Advanced secondary (years 11–13)	2	4	12		6	6	6		2	7	11	
Apprenticeship training	0	1	1		1	0	2		1	0	0	
University	0	0	10		4	1	2		2	3	4	
Employment status				< .0001				< .0001				0.0034
Paid employee	19	17	44		15	27	41		20	28	28	
Retired	3	5	5		3	4	7		4	5	7	
Unemployed, looking for work	27	39	14		66	18	4		39	28	24	
Unable to work	5	8	1		6	4	3		11	4	3	
On social benefits	0	0	1		0	0	1		0	0	0	
Full-time homemaker	45	30	30		7	47	41		25	32	36	
Student	1	2	4		2	1	3		2	2	1	
Occupation				< .0001				< .0001				0.0004
Subsistence farmer	5	2	2		1	5	2		2	3	2	
commercial farmer	0	1	1		3	0	1		3	1	1	
Casual worker	8	9	7		12	10	7		7	7	5	
Businessperson, merchant, vendor	47	47	39		63	38	42		49	49	41	
Craftsperson	7	6	5		2	10	4		4	9	6	
Civil servant	2	5	21		6	10	8		6	9	14	
Other	31	30	25		13	27	36		30	22	33	
Marital status				< .0001				< .0001				0.0094
Married/registered	43	63	66		82	58	27		59	57	60	
Cohabitating	31	19	14		2	21	51		15	21	27	
Single	1	4	12		6	3	7		4	7	5	
Divorced or separated	3	1	2		2	4	2		3	2	2	
Widowed	22	13	5		7	14	13		20	13	6	
Number of people living in the household	6(4)	6(3)	6(3)	0.2604	5(4)	6(4)	6(4)	0.3982	6(4)	6(3)	6(3)	0.0913
Main source of energy used for cooking				< .0001				0.5943				0.2089
Missing	0	0	0		0	0	0		0	0	0	
Not applicable	1	1	3		3	2	2		1	1	2	
Gas	21	40	55		41	33	37		39	34	42	
Firewood (simple format)	33	15	10		18	24	23		24	16	21	
Firewood (improved format)	17	19	12		13	16	11		13	17	12	
Coal (simple format)	17	14	9		15	13	16		15	18	14	
Coal (improved format)	10	11	12		11	12	10		9	15	9	
Oher	0	0	0		0	1	1		1	0	1	
Number of refrigerators	0(0)	0(0)	0(1)	< .0001	0(0)	0(0)	0(0)	0.1195	0(0)	0(0)	0(0)	0.7938
Living with partner (yes)	72	80	79	0.1429	82	78	75	0.0227	72	74	86	0.0002
Living with toddler (yes)	47	51	50	0.7814	55	55	45	0.0419	44	50	56	0.0005
Living with child (yes)	86	88	79	0.0037	86	84	90	0.7521	90	84	81	0.0880
Living with parents (yes)	4	5	12	0.0041	7	5	7	0.7199	4	8	10	0.0155
Living with in-laws (yes)	5	4	3	0.3583	1	3	6	0.0358	1	3	4	0.0490
Living in retirement home (yes)	1	1	0	0.1564	0	0	1	0.7231	0	0	2	0.0046
Living with other adults (yes)	11	3	3	0.0005	3	13	1	0.1124	1	3	14	< .0001
Total energy intake (kcal)	1879 (810)	1664 (889)	2280 (737)		1488 (805)	2065 (868)	1959 (686)		1321 (565)	1729 (563)	2544 (674)	
Carbohydrates (% energy)	66 (13)	63 (17)	57 (15)		71 (7)	63 (9)	46 (8)		61 (20)	59 (16)	64 (8)	
Fat (% energy)	22 (12)	24 (14)	28 (13)		17 (7)	24 (7)	37 (6)		24 (17)	27 (15)	23 (6)	
Protein (% energy)	12 (2)	12 (3)	14 (4)		11 (2)	13 (2)	16 (3)		12 (3)	13 (4)	12 (2)	

Only quintiles 1, 3, and 5 are shown. Figures are percentages, mean ± SD, or median (IQR). We calculated p-for trend using the Jonckheere trend test (non-normally distributed), linear regression (normally distributed), the Cochran–Armitage trend test (binary), and the Cochran–Mantel–Haenszel test (categorical)

To determine the variables to be included in the multivariate linear regression models, we assessed the association between factor score quintiles and sociodemographic variables (Table 5). We found no linear association for ethnic group, main source of energy used for cooking, number of refrigerators, number of housemates, living with child, and living in retirement home. The variables ‘living with partner’ and ‘living with parents’ were eliminated because they showed an association with marital status of Cramér’s V > 0.3. The remaining sociodemographic characteristics were included as independent variables in the models: age, sex, education level, employment status, occupation, International Wealth Index, marital status, and living together with a toddler/in-laws/other adults.

Table 6 shows the multivariate associations of demographic and socioeconomic variables with the dietary patterns. For the meat and egg-based dietary pattern, the explained variance was 29%. Positive associations with this dietary pattern were seen for a lower age, being male, having a higher education (reference: no formal education), being a full-time homemaker, a paid employee or being unable to work (reference: being unemployed); having a higher IWI score. Casual workers (reference: merchant); cohabitating people (reference: married); and people living with other adults were less likely to have a high score for this pattern.

**Table 6:** Multivariate linear associations of demographic and socioeconomic factors with adherence to dietary pattern scores

Factors:	Meat and egg-based dietary pattern		Fish-based dietary pattern		Starchy food-based dietary pattern	
*R*^2^	0.290		0.397		0.145	
	beta (95% CI)	*p*-value	beta (95% CI)	*p*-value	beta (95% CI)	*p*-value
Age (years)	− 0.009 (− 0.02–− 0.00)	0.0027	0.004 (− 0.00–0.01)	0.1602	− 0.007 (− 0.01–− 0.00)	0.0396
Male sex (vs. female)	0.323 (0.18–0.47)	< .0001	− 0.161 (− 0.29–− 0.03)	0.0173	0.043 (− 0.11–0.20)	0.5937
Educational level (vs. none)						
advanced secondary (years 11–13)	0.490 (0.20–0.78)	0.0011	0.098 (− 0.17–0.37)	0.4808	0.589 (0.27–0.91)	0.0004
apprenticeship training	0.819 (− 0.04–1.68)	0.0617	0.852 (0.06–1.65)	0.0357	− 0.840 (− 1.78–0.10)	0.0803
Limited secondary (years 7–10)	0.529 (0.34–0.72)	< .0001	0.379 (0.21–0.55)	< .0001	0.061 (− 0.14–0.27)	0.5579
Primary (years 1–6)	0.259 (0.11–0.41)	0.0010	0.210 (0.07–0.35)	0.0039	− 0.003 (− 0.17–0.17)	0.9686
University	1.264 (0.84–1.69)	< .0001	− 0.284 (− 0.68–0.11)	0.1585	0.250 (− 0.22–0.72)	0.2927
Employment status (vs. unemployed)						
Full-time homemaker	0.253 (0.09–0.41)	0.0023	0.848 (0.70–1.00)	< .0001	0.124 (− 0.05–0.30)	0.1707
On social benefits	1.183 (− 0.51–2.88)	0.1719	1.752 (0.18–3.32)	0.0289	− 0.698 (− 2.56–1.16)	0.4620
Paid employee	0.368 (0.20–0.54)	< .0001	1.054 (0.90–1.21)	< .0001	0.039 (− 0.15–0.22)	0.6809
Retired	0.084 (− 0.23–0.40)	0.6029	1.107 (0.81–1.40)	< .0001	0.266 (− 0.08–0.61)	0.1328
Student	− 0.143 (− 0.65–0.37)	0.5814	0.711 (0.24–1.18)	0.0031	− 0.364 (− 0.92–0.19)	0.2000
Unable to work	0.367 (0.05–0.68)	0.0238	0.326 (0.03–0.62)	0.0300	0.058 (− 0.29–0.41)	0.7430
Occupation (vs. merchant)						
Casual worker	−0.341 (−0.56–−0.13)	0.0018	0.133 (−0.06–0.33)	0.1867	0.038 (−0.20–0.27)	0.7477
Civil servant	0.185 (−0.09–0.46)	0.1879	− 0.335 (− 0.59–− 0.08)	0.0101	0.207 (− 0.10–0.51)	0.1796
Commercial farmer	0.152 (− 0.46–0.76)	0.6250	− 0.315 (− 0.88–0.25)	0.2739	− 0.612 (− 1.28–0.06)	0.0722
Craftsperson	− 0.119 (− 0.36–0.12)	0.3375	− 0.247 (− 0.47–− 0.02)	0.0321	0.084 (−0.18–0.35)	0.5352
Other	− 0.055 (− 0.21–0.10)	0.4877	0.116 (− 0.03–0.26)	0.1164	0.154 (− 0.02–0.33)	0.0793
Subsistence farmer	0.114 (− 0.24–0.47)	0.5268	− 0.057 (− 0.38–0.27)	0.7306	0.259 (− 0.13–0.64)	0.1887
IWI	0.012 (0.01–0.02)	< .0001	0.005 (0.00–0.01)	0.0033	0.003 (− 0.00–0.01)	0.1824
Marital status (vs. married/registered)						
Cohabitating	− 0.274 (− 0.42–− 0.13)	0.0003	0.832 (0.70–0.97)	< .0001	0.103 (− 0.06–0.26)	0.2124
Divorced or separated	0.001 (− 0.37–0.38)	0.9942	0.305 (− 0.04–0.65)	0.0846	− 0.202 (− 0.61–0.21)	0.3357
Single	0.045 (− 0.24–0.33)	0.7555	0.397 (0.14–0.66)	0.0028	− 0.199 (− 0.51–0.11)	0.2051
Widowed	− 0.084 (− 0.29–0.12)	0.4179	0.270 (0.08–0.46)	0.0049	− 0.299 (− 0.52–− 0.08)	0.0084
Living with toddler (vs. not)	− 0.037 (− 0.15–0.08)	0.5416	− 0.098 (− 0.21–0.01)	0.0789	0.104 (−0.03–0.23)	0.1157
Living with in-laws (vs. not)	0.210 (− 0.11–0.53)	0.2034	− 0.032 (− 0.33–0.27)	0.8362	0.165 (− 0.19–0.52)	0.3624
Living with other adults (vs. not)	− 0.394 (− 0.65–−0.14)	0.0027	− 0.503 (− 0.74–−0.27)	< .0001	1.052 (0.77–1.33)	< .0001

Beta coefficients, 95% confidence intervals (CIs), p-values, and R^2^ were calculated by linear regression

N = 935 adults living in Ouagadougou

For the fish-based dietary pattern, positive associations were discernible with female sex, higher education (reference: no education); not being unemployed (reference: being unemployed); a higher IWI score; cohabitating, being single or widowed (reference: married). Negative associations with this dietary pattern score comprised being a civil servant or a craftsperson (reference: merchant) or living with other adults. These factors explained 40% in the variance of the fish-based dietary pattern score.

Lastly, for the starchy food-based dietary pattern, we observed an explained variance of 15%, and positive associations with younger age, having obtained advanced secondary schooling (reference: no formal education), or living with other adults. Being widowed (reference: married) was negatively associated with this dietary pattern score.

### Sensitivity analysis

To test the robustness of the extracted dietary pattern scores, we performed a split-half analysis. The results are shown in Table 7. In both randomly selected halves, we observed the same three patterns with similar correlation coefficients between factors and food groups.

**Table 7:** Results of principal component analysis for the total sample and for two randomly assigned halves

	All	1. Half	2. Half
n	1000	500	500
Factors selected	3	3	3
Variance explained (%)	28.8	30.1	28.9
Variance explained by factor after rotation (%)	10.2, 9.8, 8.9	11.5, 9.6, 8.9	10.4, 10.0, 8.5
Pattern 1 |loadings|> .4	Meat and egg-based -poultry .62 -meaty mixed dishes .61 -red meat .54 -egg .54 -potatoes .45 -processed meat .42	Meat and egg-based -poultry .67 -potatoes .61 -meaty mixed dishes .60 -red meat .55 -egg .52 -fruit .43 -roots and tubers .43 -processed meat .43 -vegetables .43 -condiments .41	Meat and egg-based -meaty mixed dishes .66 -poultry .54 -red meat .53 -egg .52 -dairy .47 -condiments .46 -vegetarian mixed dishes (−0.43)-maize-based (−0.48)
Pattern 2 |loadings|> .4	Fish-based -fish .71 -palm oil .70 -condiments .63 -red meat .55 -meaty mixed dishes .50 -vegetables .47 -nuts and seeds .44	Fish-based -palm oil .79 -fish .70 -red meat .55 -meaty mixed dishes .50 -condiments .43 -rice, pasta, corn (−0.44) -maize-based foods (−0.47)	Starchy food-based -vegetables .57 -nuts and seeds .53 -potatoes .53 -condiments .53 -roots and tubers .49 -rice, pasta, corn .48 -legumes .46 -fruit .46
Pattern 3 |loadings|> .4	Starchy food-based -rice, pasta, and corn .61 -roots and tubers .48 -maize-based foods .47 -condiments .46 -vegetables .46 -nuts and seeds .44 -cakes and sweets .44 -legumes .43 -fruit .43 -potatoes .42	Starchy food-based -cakes and sweets .63 -condiments .62 -vegetables .53 -nuts and seeds .52 -rice, pasta, corn .49 -other oils .44 -maize-based foods .42 -legumes .41	Fish-based -palm oil .70 -fish .53 -condiments .48 -red meat .48 -vegetables .43 -meaty mixed dishes .42 -coffee, tea (−0.43) -rice, pasta, corn (−0.43) -dairy (−0.45)

In order to examine the robustness of the derived dietary patterns, we conducted a sensitivity analysis, performing PCA separately by settlement type. The resulting factors with associated food groups are shown in Table 8. The pattern scores in the formal settlements corresponded well to the three patterns from the total sample, also representing a meat- and egg-based pattern, a fish-based pattern, and a pattern with high loadings of starchy foods. In the informal settlements, we also found patterns with similar interpretation: a fish- and meat-based pattern, a pattern with high loadings for starchy staples and other plant-based foods, and a pattern with a combination of animal-based foods. Owing to the similarities of dietary pattern scores between settlement types, it was reasonable to use the PCA results of the total sample for the subsequent association analyses.

**Table 8:** Results of principal component analysis for the total sample and separately for settlement types

	All	Formal settlements	Informal settlements
n	1000	501	499
Factors selected	3	3	3
Variance explained (%)	28.8	29.4	29.7
Variance explained by factor after rotation (%)	10.2, 9.8, 8.9	11.2, 10.0, 8.2	11.9, 9.1, 8.5
Pattern 1 |loadings|> .4	Meat and egg-based -poultry .62 -meaty mixed dishes .61 -red meat .54 -egg .54 -potatoes .45 -processed meat .42	Meat and egg-based -poultry .65 -potatoes .61 -meaty mixed dishes .54 -egg .53 -fruit .50 -red meat .48 -roots and tubers .43 -vegetables .42	Fish-/meat-based - palm oil .78 -fish .74 -Meaty mixed .69 -red meat .69 -condiments .63 -processed meat .40
Pattern 2 |loadings|> .4	Fish-based -fish .71 -palm oil .70 -condiments .63 -red meat .55 -meaty mixed dishes .50 -vegetables .47 -nuts and seeds .44	Fish-based -condiments .79 -fish.59 -vegetables .58 -red meat .53 -other oils .53 -meaty mixed dishes .46 -nuts and seeds .44 -cakes and sweets .41 -coffee tea (−0.42)	Starchy staples and plant-based -vegetables .51 -cakes and sweets .51 -maize-based .50 -nuts and seeds .50 -condiments .48 -rice, pasta, and corn .44 -legumes .44 -roots and tubers .44 -other oils .42 -potatoes .42
Pattern 3 |loadings|> .4	Starchy food-based -rice, pasta, and corn .61 -roots and tubers .48 -maize-based foods .47 -condiments .46 -vegetables .46 -nuts and seeds .44 -cakes and sweets .44 -legumes .43 -fruit .43 -potatoes .42	Starchy food-based -rice, pasta, corn .69 -maize-based .61 -legumes .50 -palm oil (−0.59)	Animal-based -dairy .56 -poultry .48 -egg .45 -coffee, tea .44 -meaty mixed dishes .43 -fruit .42

## Discussion

### Key findings

The purpose of this study was to identify common dietary patterns and to describe their sociodemographic factors of adherence in a representative sample from urban sub-Saharan Africa. The most frequently consumed food groups included plant-based staples (rice, pasta, and corn; maize-based foods; vegetables; white bread and cereals); animal-based products (egg, poultry, dairy products, red meat, fish) were rarely consumed. We found three distinct dietary patterns: a meat and egg-based pattern that was strongly correlated with poultry, meaty dishes, red meat, and eggs; a fish-based pattern characterized by fish, palm oil, condiments, and red meat; and a starchy food-based pattern that correlated with rice, pasta, and corn, roots and tubers, maize-based foods, and condiments. Adherence to the meat and egg-based pattern was associated with younger age, male sex, higher education, and an advanced economic situation; the fish-based pattern was associated with female sex, a higher education, and a higher economic status; whereas the starchy food-based pattern was related with younger age, and sharing a house with other adults.

### Study population

A noteworthy finding pertaining to the study population is the overrepresentation of women of 64.3% compared to a balanced men to women ratio of 100.6:100 in the total HDSS population [21]. This is a common phenomenon in surveys conducted in the homes of participants. One sensible explanation is that, on average, women might spend more time at home than men [29]. However, the sex ratio in the present study was very similar between the different settlement types, suggesting that this effect had a similar influence, regardless of the neighbourhood.

### Dietary habits and the nutrition transition

According to Popkin’s framework of the nutrition transition, shifts in dietary behaviour are associated with economic development, demographic changes and changes in the prevalence of certain diseases or risk factors. In the following section, the results of this study are put into context of the stages in this model, ranging from stage 3 (high consumption of fibre, starchy foods, low consumption of fat and meat) through stage 4 (high in fat, sugar, and processed foods) and up to stage 5 (more health-conscious, consisting of reduced intake of fat and sugar in favour of more fruits and vegetables). [2]

In this study population, absolute intakes of all macronutrients and energy seemed to be much lower in informal settlements as compared to formal settlements. This relative difference between settlement types suggests that the population in informal settlements consumes less food overall. The intake of macronutrients relative to energy intake was similar for the different groups analysed. In the total study population, the median carbohydrate intake of 62 energy% slightly exceed the reference intakes suggested by the European Food Safety Authority of 45–60 energy% [30]. Conversely, total fat intake and protein intake were lower in the present study group than the recommended intakes [30]. In summary, the low intake of protein and fat with high intake of carbohydrates suggests that this population is at an early stage of the nutrition transition (stage 3), holding true not only for the general study population but also for the different settlement types analysed [2].

This interpretation is further corroborated by the predominating foods in our survey. These were starchy staples based on maize and other cereals, and vegetables. At the same time, the consumption of meat, processed meat, added fats, and sweet spreads was rather low.

Finally, the combination of foods consumed, as identified by PCA, and the associated sociodemographic factors of adherence underpin the notion that enhanced economic status leads to modernized dietary practices (referring to higher intake of refined carbohydrates, processed foods, red meat). Certain groups are more inclined to eat a diet richer in animal-based products (the meat- and egg-based dietary pattern) than others. Here, younger people, men, better educated subjects, and those with in a better economic situation have a higher probability of adhering to this diet. Similarly, Table 3 shows that people from formal settlements have higher scores for this dietary pattern. This might suggest that men, better educated people and those in a better economic situation might be the groups that are already on their way to the next stage of the nutrition transition (stage 4 according to Popkin).

### Factors of adherence to dietary patterns in sub-Saharan Africa

Compared to other studies from urban sub-Saharan Africa, we found an unusually homogenous meat and egg-based pattern. Nkondjock et al. [31] found a similar pattern made up mostly of bush meat, poultry and red meat in 541 Cameroonian adults. Other studies, however, found meat-heavy dietary patterns that also showed strong association with fish and wheat-based foods [24] or with nuts and legumes [32]. Nkondjock et al.’s meat-based pattern was associated with older age, higher energy intake and BMI, as well as low educational level. This contradicts our finding that the meat- and egg-based pattern was associated with younger age and a high educational level. One reason for this might be that the population studied by Nkondjock et al. (members of the Cameroonian military) overall had a higher socioeconomic status than the population we studied. Whereas in our study population, animal-based products might be hard to access for a large part of the group for economic reasons, this might not have been a limiting factor in Nkondjock et al.’s study. The dietary pattern that included meat and fish in the study by Galbete et al.[24] was associated with living in urban Ghana, as opposed to residing in Europe or rural Ghana, with a higher educational level, male sex, younger age; and more physical activity. This corresponds well to the association we found regarding male sex and younger age being associated with stronger adherence to the meat- and egg-based pattern. Obasohan et al. [32] sought to determine the association of dietary patterns with high blood pressure in Nigerian civil servants but did not identify the meat, nuts, and legume pattern as a significant risk factor.

Mank et al. [33] also found a fish-based dietary pattern in 514 children from rural Burkina Faso. In contrast to the pattern we found, however, it was also strongly associated with maize-based foods, whereas our fish-based pattern had a negative association with maize-based foods.

The starchy food-based pattern in this study corresponds best to the “traditional” patterns found in many studies, most frequently consisting of cereals, grains, roots and tubers, corn, nuts and legumes, fermented foods, and staples, such as tô (the national dish of Burkina Faso consisting of corn, millet or sorghum flour) [10, 13, 24, 34]. One study conducted in 2010 in 330 households in Ouagadougou found one “traditional” dietary pattern through cluster analysis, which was characterized by high intakes of leafy vegetables and local cereals and comprised 71% of the population as opposed to 29% classified in an “urban” dietary pattern (higher intake of fat and sugar). Similar to our findings, the traditional dietary pattern had positive associations with the female sex, lower income, and lower education. [10] Similarly, two studies from Ghana also found associations between “traditional” dietary patterns, lower education, lower income, and rural populations [24, 34]. Comparable combinations of foods to the “traditional” dietary patterns are sometimes also summarized as “health conscious” (in this study characterized by high intakes of foods such as vegetables, fruits, roots and tubers, while being low in snacks and sweetened foods) [35]. Classifying such patterns as health conscious, however, is difficult using this methodology, as important factors to the healthfulness of a diet (e.g., dietary variety) are not considered. This is one of the reasons why we opted to name the dietary patterns we found in a more descriptive manner, mentioning the most highly loading food groups.

Unlike previous literature, we did not identify a “sweets” or “snacking” pattern, characterized by high intakes of sweetened snacks, soft drinks, and similar foods [12, 13, 35, 37]. One reason for this might be the fact that many of the studies identifying this pattern were conducted in children or adolescents and the pattern seems to correlate with younger age [37], whereas our study included only adults starting from the age of 25. In addition, the lack of a snacking pattern in our study supports the conclusion that our study population is still at an early stage of the nutrition transition.

### Strengths and limitations of this study

This study stands out through its large sample size of 1,000 participants, drawn from a representative sample within the only urban HDSS in Western Africa, including formal and informal settlements. Using PCA to derive dietary patterns, we did not have to rely on “a-priori” reference values. Furthermore, through this method we were able to take into account the whole diet, not just single nutrients. In contrast to cluster analysis (CA), PCA yields continuous variables as results, suitable for subsequent regression analyses. Unlike CA, PCA does not rely on a reference group, thus not limiting statistical power [38]. Moreover, PCA assigns a score to each participant for every dietary pattern unlike CA which yields uneven, mutually exclusive groups, which might limit interpretability [39]. The detailed, region-specific food-frequency questionnaire allowed to cover a wide range of foods and dishes common in the target population.

Food-frequency questionnaires tend to underestimate energy intake due to the closed list of food items. Therefore, the derivation of absolute nutrient intakes may not be accurate. However, on a population basis and for between-group comparisons, FFQ data can be useful to rank participants according to their nutrient intakes. Energy intake, for example, might be underreported by as much as 20% [40]. Furthermore, the AFPQ has not been validated in a Burkinabe population yet. For this reason, we focused on relative intergroup differences, rather than using absolute intake values. Additionally, food-frequency questionnaires do not take into account the mode of preparation of the foods which might impact nutrient uptake. Further limitations of our study include the subjective decisions involved in principle component analysis, such as deciding the number of components to retain, the rotation method, and setting cut-off values for factor loading scores. This might limit comparability with other studies. While the sample was designed to be representative and sample size was comparatively large, the validity of our results might be impacted by response bias due to some sociodemographic groups being more willing to participate, as demonstrated by the disbalanced sex distribution of the respondents. Also, in retrospective dietary assessment, recall bias can distort the findings, when some groups respond systematically differently than others.

### Public health implications

Interventions with the goal of promoting a more healthful diet that can profit from the insights gained in this study range from the individual level to interventions that target the general food environment.

On the individual level, nutrition education interventions have been used successfully to improve nutrition knowledge and physical activity level [41, 42]. Our study suggests that educational interventions in Ouagadougou should focus on promoting moderate intakes of starchy foods, while increasing the intake of protein sources. However, interventions should also highlight the risks associated with high intakes of red meat and promote adoption of alternative protein sources (poultry, pulses and other plant-based protein sources). As the nutrition transition progresses, the population should be encouraged to maintain the current habits of low snacking, and avoiding sweets, alcohol, and red meat. Special attention should be paid to the role of men, younger and more affluent population groups, as those appear to have a stronger inclination for meat- and egg-based dietary patterns. Those groups could be the first to move away from traditional diets and thus, becoming at risk of adopting unhealthy diets.

Interventions that work on the food environment level could improve the availability and desirability of certain foods, developing value chains, accompanied by community work [43]. Alternative sources of protein (other than red meat) should be made available and promoted through marketing campaigns. On the policy level, governments can influence the risk of nutrition-related noncommunicable diseases through interventions such as restricting marketing for unhealthful foods (e.g., sweets and snacks) to prevent those from gaining more popularity in this population), funding research on specific risk factors, and improving monitoring and evaluation of adverse consequences [44]. Taxing highly processed foods can be a means of influencing both obesity, as well as undernutrition [45].

### Research Implications

With the aim of understanding determinants of a healthy diet, it is necessary to conduct further research into the interpersonal, environmental and policy levels of influence, as those upstream factors of individual decision making are still poorly understood [46]. With the knowledge of *how* people eat, the question opens up *why* they choose to do so and what might be barriers on the interpersonal, environmental, and policy levels that prevent individuals from choosing a more healthful diet. Future research should be dedicated to understanding the health consequences of different dietary patterns. It will be important to understand the relationship between meat-based dietary patterns and cardiovascular diseases or type 2 diabetes. Another important factor that might be target of future research in similar contexts is the role of food processing. Using food processing classification systems, such as the NOVA [47] system to classify the food items present in the dietary patterns found could be helpful in understanding the progress of the nutrition transition.

Our study provides a valid starting point to generate in-depth understanding about the nature of the nutrition transition in Ouagadougou and other urban areas in sub-Saharan Africa. In the future, repeated measurements are desirable to evaluate the changes in dietary behaviour over time [48]. This might allow for new insights into the dynamics of diet and for predictions regarding future trends. Health and demographic surveillance systems, such as the one in Ouagadougou, would be ideal for such studies, offering the infrastructure and representative samples of urban populations.

## Conclusions

We conclude that in this population from urban Burkina Faso, distinct dietary patterns are discernible that vary according to economic status, age, and educational attainment. A dietary pattern high in animal-based products is prevalent among young, affluent and well-educated individuals. This contrasts with a diet high in starchy foods and associated with bigger households and lower educational attainment. Interestingly, the differences between sociodemographic groups seem to be minor, with an overall low intake of processed foods or meat, and high intakes of starchy foods. In general, this study population seems to be at an early stage of the nutrition transition (stage 3) and health consequences need to be investigated.

These findings imply that a general reduction of starchy roots and tubers with increased intake of plant-based protein sources should be encouraged. At the same time, important nuances in dietary practices that stem from differences in socioeconomic characteristics highlight the need for target-group-specific interventions in this area.

### Supplementary Information

**Additional file 1**: STROBE-nut checklist.

## Data Availability

The datasets used and analysed during the current study are available from the corresponding author on reasonable request.

## Abbreviations

AFPQ: African Food Propensity Questionnaire
CA: Cluster Analysis
CI: Confidence Interval
HDSS: Health and Demographic Surveillance System
IWI: International Wealth Index
PCA: Principal Component Analysis
SD: Standard Deviation
SSA: Sub-Saharan Africa
